# Taming the Boys for Global Good: Contraceptive Strategy to Stop Malaria Transmission

**DOI:** 10.3390/molecules25122773

**Published:** 2020-06-16

**Authors:** Ryan Choi, Samantha A. Michaels, Emmanuel C. Onu, Matthew A. Hulverson, Aparajita Saha, Morenike E. Coker, Janis C. Weeks, Wesley C. Van Voorhis, Kayode K. Ojo

**Affiliations:** 1 Center for Emerging and Re-Emerging Infectious Diseases (CERID), Division of Allergy and Infectious Diseases, Department of Medicine, University of Washington, Seattle, WA 98109, USA; ryan6424@uw.edu (R.C.); samich@uw.edu (S.A.M.); mhulv@uw.edu (M.A.H.); apsaha@uw.edu (A.S.); wesley@uw.edu (W.C.V.V.); 2 Department of Pharmaceutical Microbiology, Faculty of Pharmacy, University of Ibadan, Ibadan, Nigeria; emmanuels114@gmail.com (E.C.O.); morencoker2002@yahoo.com (M.E.C.); 3 Department of Biology, University of Oregon, Eugene, OR 97403, USA; jweeks@uoregon.edu

**Keywords:** malaria, *Plasmodium* spp., transmission reducing interventions, bumped kinase inhibitors, calcium-dependent protein kinase inhibitors

## Abstract

Transmission of human malaria parasites (*Plasmodium* spp.) by *Anopheles* mosquitoes is a continuous process that presents a formidable challenge for effective control of the disease. Infectious gametocytes continue to circulate in humans for up to four weeks after antimalarial drug treatment, permitting prolonged transmission to mosquitoes even after clinical cure. Almost all reported malaria cases are transmitted to humans by mosquitoes, and therefore decreasing the rate of *Plasmodium* transmission from humans to mosquitoes with novel transmission-blocking remedies would be an important complement to other interventions in reducing malaria incidence.

## 1. Introduction

**Figure 1:**
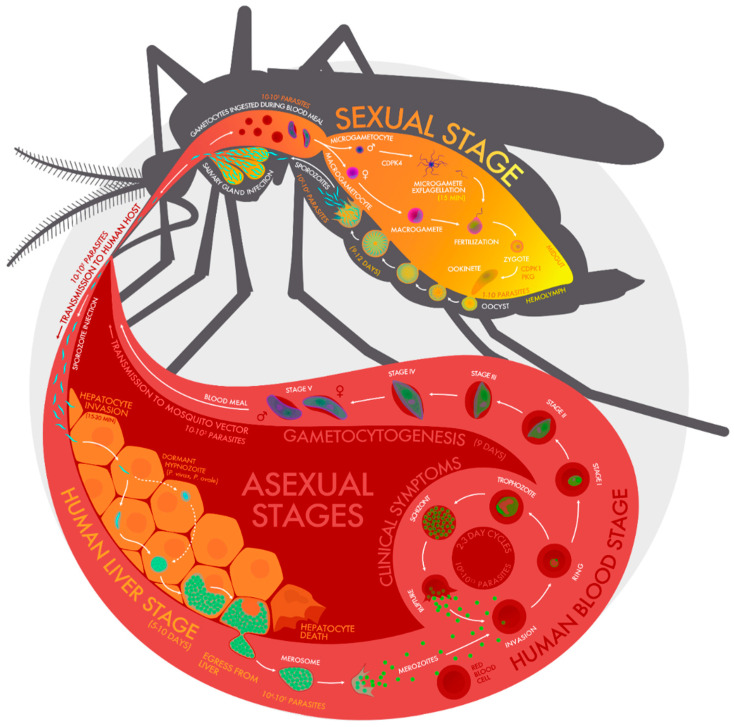
Diagram of the malaria parasite life cycle showing asexual stages (mammalian host); gametocytes in blood, gametocytes in mosquito gut with exflagellation of microgametes, fertilization of macrogamete, formation of ookinete, movement across mosquito gut to form oocysts, oocysts rupture to form sporozoites that mature in the mosquito salivary glands, sporozoites injected into a human, and movement to liver hepatocytes, replication, and rupture releasing thousands of merozoites that can infect red blood cells and replicate via asexual life cycle. The approximate number of parasites and length of each stage of malaria parasite development are shown.

Malaria remains a major cause of morbidity and mortality, afflicting over two hundred million people annually [1]. In humans, clinical malaria is caused by five different species of the *Plasmodium* genus, namely *P*. *falciparum* (*Pf*), *P*. *vivax*, *P*. *malariae*, *P*. *knowlesi*, and *P*. *ovale* [2]. The disease is mainly transmitted to humans through the bites of female *Anopheles* mosquitoes, which inject *Plasmodium* sporozoites into the bloodstream [3]. These infected individuals, often without symptoms, continue to transmit malaria parasites back to vector hosts. The sporozoites quickly travel to the human liver where they multiply asexually for approximately 10 days before being released in vesicles called merosomes. The merosomes, then, travel to the lung in vesicles for subsequent release into the bloodstream as merozoites. The merozoites invade red blood cells and repeatedly multiply asexually to initiate the human clinical stages characterized by symptoms including fever, myalgias, and pernicious syndromes sometimes leading to death. A small number of merozoites that have infected blood cells develop first into immature gametocytes (sexual stages), which develop further into mature sexual stages called microgametes (male) and macrogametes (female). Both types are transmitted to mosquitoes during subsequent bites of the human host (Figure 1).

In mosquitoes, exflagellation of the microgamete and fertilization of macrogamete initiate the development of new parasites in the mosquito. Exflagellation is the visible outcome of the maturation and activation of male gametocytes into sperm-like, male gamete cells needed for fertilization of the female gametes inside the mosquito gut [4]. In addition to the extended presence of gametocytes infectious to mosquitoes, malaria deaths are mostly associated with *P*. *falciparum* infections. In that sense, discussions of malaria eradication skew toward effective control of *falciparum* malaria. Overall, this article focuses on strategies to reduce malaria parasite transmission, specifically the development of inhibitors that target stages of the *Plasmodium* life cycle that transpire in mosquitoes. Targeting drug treatments to mosquitoes has been advocated as a promising but underutilized approach for malaria control [5]. For the purpose of this review, we focus on drug development efforts aimed at one specific portion of the *Plasmodium* life cycle in mosquitoes, i.e., the early sexual stage.

## 2. Current State of Affairs

Malaria control has traditionally employed the following two main fronts: (1) effective management of clinical cases and (2) control of the *Anopheline* mosquito vector. Case management has relied largely on antimalarials classified here on the basis of their chemical structures. The major classes include aryl amino alcohol compounds (quinine, quinidine, lumefantrine, halofantrine, and mefloquine); 4-aminoquinolines (chloroquine, amodiaquine, and piperaquine); 8-aminoquinolines (primaquine and tafenoquine); naphthoquinones (atovaquone); antifolate compounds (sulfadoxine-pyrimethamine, proguanil, and chlorproguanil); and artemisinin compounds (artemisinin, dihydroartemisinin, artemether, and artesunate) [6,7,8,9,10]. Artemisinin-based combination therapy (ACT), the first-line treatment in many endemic regions for uncomplicated malaria, removes a vast majority of the malaria parasites and rapidly alleviates symptoms [11]. ACT has been a life-saving innovation, using the basic principle that the short half-life, but highly potent, artemisinin derivative delivers a rapid reduction in parasite biomass, while an inherently less active, but more slowly eliminated, partner drug eliminates the remaining parasites [12].

While ACT is an effective strategy for the first front of malaria control, artemisinin kills early stages (I–III), but not later stages (IV and V) of gametocytes. Following ACT treatment and abatement of clinical symptoms, gametocytes can remain viable in the human blood circulation, permitting continued transmission to mosquitoes [13]. Thus, parasite transmission from humans to vectors is a critical checkpoint in the infection cycle that must be targeted as one component of controlling and eventually eliminating malaria.

The second front of malaria control aims to manage the *Anopheline* mosquito vector through removal of breeding sites, use of insecticides, and prevention of contact with humans via screens and bed nets, particularly ones that are impregnated with insecticides [14]. A drug-based example undergoing clinical investigation is the use of mass drug administration of ivermectin to humans to control mosquitoes [15]. The downsides are that ivermectin has the potential to disrupt ecosystems by impacting mosquito populations, and widespread use of ivermectin could promote resistance in other important human and animal parasites. Considerable improvements in the control and management of malaria have come in recent years through programmed interventions, and novel ideas that could lead to even more effective outcomes are currently being debated.

## 3. New Malaria Transmission-Blocking Strategies Are Greatly Needed

Despite these gains, malaria continues to cause a staggering burden of disease in regions of Southeast (SE) Asia, and East and Sub-Saharan Africa, especially among infants and children [16]. Efforts for disease eradication have been stymied by numerous challenges, such as increasing mosquito resistance to long-lasting insecticidal nets (LLINs) [17], emergence of *Plasmodium* resistance to artemisinin [18], and a lack of a completely protective vaccine [19]. While significant declines in human malaria prevalence and mortality rates in recent years can be attributed to the use of ACT [20,21,22], ACT still allows for transmission of residual *P. falciparum* gametocytes back to vector hosts for 6 to 15 days after therapy, as compared with ≥28 days in untreated individuals [23]. In other malaria parasite strains, such as *P*. *vivax*, the process of gametocyte transmission to mosquitoes is more efficient relative to *P*. *falciparum*. *P*. *vivax* gametocytemia is commonly detectable at higher densities early in the course of the infection, but the duration of carriage of the gametocytes is considerably shorter after treatment as compared with that of *P*. *falciparum* [11,24,25]. The recent emergence of artemisinin resistance in parts of Southeast Asia [26] is also a disturbing global health concern that underscores the need for immediate interventions to prevent parasite transmission. However, it is important to distinguish between artemisinin resistance and ACT resistance. Resistance to ACT is defined by recrudescence of the infecting strain, usually within 28 days post treatment. Artemisinin resistance is characterized by delayed clearance of parasites, which can lead to poor malaria treatment outcomes. ACT resistance in Africa primarily results from partial resistance to the partner drug. In SE Asia, artemisinin resistance contributes to ACT resistance [27] and is defined by the presence of parasitemia 3 days post treatment [28,29]. Selection for resistance to the partner drug by residual and reinfecting parasites probably follows artemisinin elimination, which occurs quickly due to its short half-life [28,29]. As a result, it is anticipated that partner drug resistance should occur more frequently in zones where artemisinin resistance is found. A broad-based strategy is required to reduce or prevent these forms of acquired resistance from developing and spreading in order to preserve the benefits of ACTs.

Development of effective malaria vaccines remains a challenge, and progress has been hindered by immune evasion mechanisms of the blood stage of *Plasmodium* spp. and the need for sterilizing immunity for vaccines directed at the liver stage [30]. Many studies have concluded that even with years of exposure to intense malaria transmission, individuals living in endemic areas are unable to acquire sterile immunity to *P*. *falciparum* infection, and these individuals continue to transmit malaria to mosquitoes often in the absence of malaria symptoms [31].

Finally, mosquitoes have emerged around the world that are resistant to insecticides which are widely employed for residual domicile spraying and for impregnating bed nets [32]. Given the direct association between malaria disease burden and *Anopheles* mosquito bites [33], this resistance threatens malaria control efforts worldwide. Therefore, it is an urgent priority to develop newer and more effective methods of intervention as a complement to existing methods for better control and prevention of malaria. Efforts to prevent and control malaria need to continuously introduce intervention strategies that would alter the dynamics of transmission.

Interventions need to be tailored to local transmission dynamics [34,35]. This is because transmission reducing interventions (TRIs) can be effective malaria control and eradication tools in low-transmission populations in the absence of other efforts, but can fail in high-transmission regions [36]. TRIs need to be combined with other control strategies to provide a tailored, multi-pronged attack in order to address both low- and high-transmission environments and to reduce the likelihood of genetic resistance developing in parasites.

## 4. New Opportunities to Prevent Malaria Transmission: Drug-Based Infection Blockers That Act in the Mosquito Have Advantages

As part of an integrated approach to malaria control and eradication, there is increasing interest in targeting the *Plasmodium* life cycle stages that occur in mosquitoes. By preventing establishment of an infection, or preventing successful development of *Plasmodium* progeny within mosquitoes, the parasite life cycle can be terminated. One example of a specific, broad-based approach to break the transmission pathway includes vaccines that target gametocytes. When anti-gametocyte antibodies and gametocytes are ingested by mosquitoes in blood meals, the antibodies block malaria parasite development in the insect host [37]. The effective gametocyte target antigens are not displayed in humans, but rather become exposed in the mosquito gut, where the antibodies have their role in blocking infection. The development of effective mosquito infection-blocking vaccines is challenging due to the fact that the target resides in a host which is different from the individual receiving the vaccine. Antibodies targeting a parasite gene expressed in the mosquito stage of development may not get the necessary immune boost for long-term viability or even enough for a season, since the target may not be expressed adequately in humans [37]. Thus, it is anticipated that widespread and frequent revaccination with gametocyte-directed vaccines would be necessary to control malaria infection of mosquitoes.

The logistical shortcomings of a transmission-blocking vaccine could be overcome by using small molecule agents that act inside the mosquito. These agents could be formulated to provide transmission-blocking properties via oral administration to humans, which would be a less invasive method of administration than a parenteral vaccine. Before the appearance of resistant *Plasmodium* strains, successful suppression of clinical malaria incidence with weekly prophylactic doses of pyrimethamine alone or with sulfadoxine provided an example of drugs used for malaria control [38,39,40,41]. An advantage of targeting parasite development in mosquitoes versus humans includes the decreased probability of selecting for drug resistance. Billions of asexual parasites are present in a human malaria patient as compared with only 1 to 10^5^ gametocytes estimated to be ingested by a mosquito during a blood meal (Figure 1) [42]. Therefore, there is a much larger pool of *Plasmodium* genetic variants in the human upon which drugs can act via natural selection as compared with the smaller number of parasites present in mosquitoes. Gametocidal drugs, such as primaquine, that act only in humans have had limited transmission-blocking success. Primaquine, especially when added to ACTs, reduces gametocyte carriage but does not completely eliminate transmission from humans to mosquitoes [43,44,45]. Reappearance of infection can be linked to a residual population of maturing gametocytes. The use of primaquine as a gametocidal agent is also limited due to the risk of hemolysis with prolonged administration in patients with a significant enzymatic deficiency of glucose-6-phosphate dehydrogenase (G6PD) [46].

Given malaria’s high prevalence and the limited available resources to fight it in endemic regions, interventions must be based on carefully selected molecular targets or stages of development that will result in the safest and most effective transmission-blocking results. Several stages of *Plasmodium* development in mosquitoes could be potential targets (Figure 1). Although male and female gametocytes play different roles in achieving efficient establishment of mosquito infection, earlier studies have described significant sex-related differences in sensitivity to small molecule treatment. These studies demonstrated that developmental pathways of male gametocytes are more vulnerable to small molecule-based transmission-blocking strategies than female gametocyte pathways [47]. Mitogen-activated protein kinase 2 (MAP2) can be a good transmission blocking target, for example, and is highly conserved in major protozoans. MAP2 was shown to play an essential role in critical cellular processes including cell survival. In *Plasmodium* spp., MAP2 has been found to be responsible for the development of male gametes during exflagellation in the mosquito gut [48,49]. Similarly, high concentrations of protease inhibitors 1,10-phenanthroline, TPCK, and TLCK were found to be effective in inhibiting *P*. *berghei* exflagellation center formation, suggesting that proteolysis is important in sexual development and could be further explored as a potential antagonizable pathway for transmission-blocking drug development [50]. Bacitracin inhibition of a male-specific *Plasmodium* protein disulphide isomerase could also be considered in future drug development efforts focused on exploring agents that block malaria transmission to mosquitoes [51]. A potential drawback for advancement to clinical development is the safety and tolerability of some of the inhibitors mentioned above.

In the following sections, we will discuss how a series of bumped kinase inhibitors (BKIs) can disrupt the transmission of malaria from humans to mosquitoes by targeting the early sexual stages of male gametocytes through treatment of either humans or mosquitoes.

## 5. Bumped Kinase Inhibitors (BKIs) as Malaria Infection Blockers in Mosquitoes: Mechanism of Action

*Plasmodium* calcium-dependent protein kinase 4 (CDPK4) has previously been shown to be essential for exflagellation of microgametes, sexual reproduction, and infection of the mosquito vector. This enzyme target is highly conserved across all *Plasmodium* species known to cause human malaria [52,53]. Inhibition of *Plasmodium* CDPK4 by BKIs blocks the differentiation and exflagellation of male gametes (Figure 1), thereby halting the development process [54,55,56]. BKIs can be likened to chemical condoms or male birth control pills that stop the development of *Plasmodium* parasites in the mosquito and prevent subsequent transmission to humans [54,55,56,57]. Lead BKIs are 4-amino-3-phenylpyrazolo [3,4-*d*] pyrimidine scaffolds: a series of type 1 protein kinase inhibitors that make similar hydrophobic and hydrogen-bonding interactions as the adenine ring of ATP, but are unique in that they are derivatized at the C3 position with bulky aromatic groups [54,55,56]. These BKIs, along with another group of compounds based on an alternative scaffold, 5-aminopyrazole-4-carboxamide analogues, selectively inhibit *P. falciparum* CDPK4 (*Pf*CDPK4) [57].

**Figure 2:**
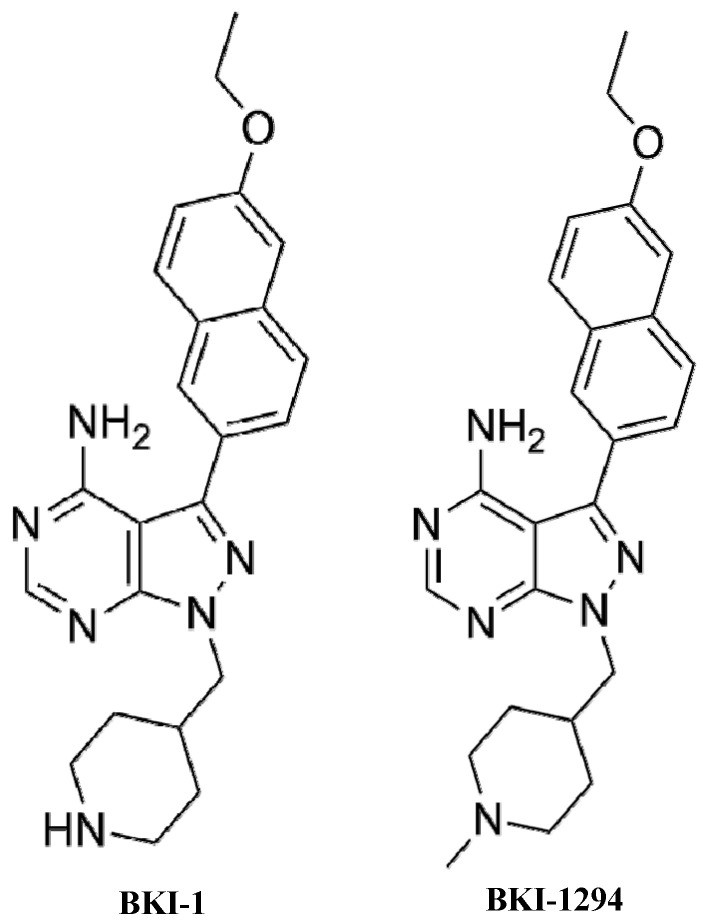
Chemical structure of BKI-1 and BKI-1294.

**Figure 3:**
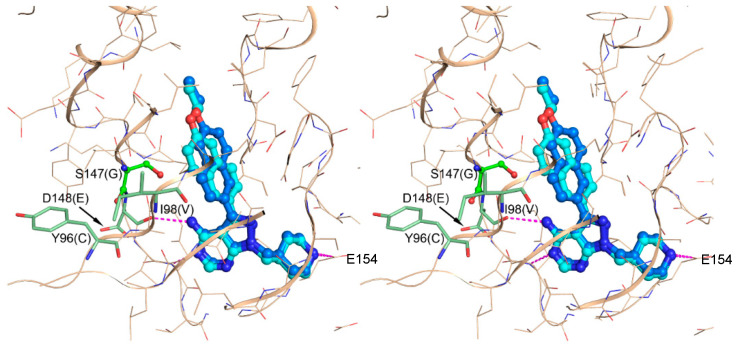
Stereo view of two possible binding modes of BKI-1 with *Pf*CDPK4 are predicted based on the crystal structure of the BKI with *Tg*CDPK1. BKI-1 is shown as blue and cyan ball-and-sticks. In both binding modes, the scaffold makes conserved hydrogen bonds to the hinge region common to most ATP analogs and the methylpiperidine substituent hydrogen bonds to E154 (as seen with the equivalent *Tg*CDPK1 E135 in crystal structures of the enzyme in complex with different ”bumped” kinase inhibitors (BKIs) that contain this R2 group). Hydrogen bonds are depicted as magenta dashed lines.

Selectivity of BKIs for *Pf*CDPK4 relative to mammalian kinases results from the unique topography of the ATP-binding domain in *Pf*CDPK4, which lacks a bulky gatekeeper residue side chain that is present in most mammalian kinases. With their bulky aromatic C3 substitution, BKIs primarily target the large hydrophobic pocket created by the presence of a serine residue (S147) at the gatekeeper position of *Pf*CDPK4. Although the *Pf*CDPK4 serine gatekeeper residue is a little larger than the glycine of *Toxoplasma gondii* CDPK1 (*Tg*CDPK1), the enzymes’ ATP binding pockets/active sites are remarkably conserved overall. Therefore, optimization for activity against *Pf*CDPK4 that led to BKI-1 and BKI-1294 (Figure 2), as previously discussed [54,55,56], was guided by iterative improvement using co-crystallization of recombinant *Tg*CDPK1 with BKIs with differing chemical functional groups on the core scaffold. Therefore, we modeled the active site of *Pf*CDPK4 based on the active site of *Tg*CDPK1 bound with BKI-1 to show how the compound interacts with residues, including the one in the gatekeeper position (Figure 3) [54,56]. The ethoxynaphthyl “bump” of BKI-1 occupies the hydrophobic pocket behind the S147 gatekeeper residue. Some BKIs (including BKI-1 and BKI-1294) have chemical modifications of the R2 groups, like a piperidine substituent, which ensure an additional conserved hydrogen bond interaction with residues (in this case, glutamic acid 154 [E154]) in the ribose pocket within the ATP binding cavity [55,58] (Figure 3). At higher concentrations, BKIs also inhibit CDPK1 and cGMP-dependent protein kinase (PKG) activities, which are needed for exflagellation and, more importantly, the zygote-to-ookinete transformation [59,60,61]. Motile ookinetes penetrate the gut wall to form oocysts, where sporozoites form to be released into the hemocoel by rupture of the oocyst and migrate to the salivary glands for transmission [62]. Thus, dual potent inhibition of CDPK1 and PKG by BKIs disrupts zygote-to-ookinete stage development.

## 6. Methods for Drug Delivery

### 6.1. BKI Delivery to Mammalian Hosts as a Single Agent

BKIs based on the pyrazolopyrimidine scaffold (e.g., BKI-1 and BKI-1294) [54,55,56] which block infection of mosquitoes by *Plasmodium* parasites, are orally available in mammals, and have low toxicity in mice [54,55]. The inability of mosquitoes to develop infection after feeding on mouse blood containing *Plasmodium* parasites and BKIs clearly demonstrates that formulation of these compounds as antimalarial drugs could be effective as transmission-blocking agents. BKI analogues that inhibit *Pf*CDPK4 at 1 to 100 nanomolar IC_50_s (50% inhibitory concentrations) concurrently prevent the exflagellation of *P. falciparum* with EC_50_ (50% effective concentration) values of 50 to 1000 nM [54,55,56]. The compounds have bioavailability and are easy to synthesize from available materials. However, widespread cases of non-adherence to medication is a persistent challenge to effective control of malaria [63]. This challenge can be addressed with effective formulations to reduce the number of doses needed to achieve the desired outcome. Several examples of formulations that would encourage widespread compliance and effective coverage could include gummy bears in the shape of mosquitoes as single daily or bi-weekly doses, transdermal patches that could be changed weekly or monthly, or low doses in widely used food supplements such as table salt. Edible salt fortified with the drug diethylcarbamazine was employed as a financially sustainable means for eliminating lymphatic filariasis [64]. All options would require smart formulation to avoid overdosing, when toxicity limits are better understood. Importantly, the finding that transmission-blocking occurs after mice ingest BKIs indicates that the compounds are stable enough in mammals to be active when delivered to mosquitoes through a blood meal.

### 6.2. Co-Formulation of BKIs with ACT

Artemether-lumefantrine (AL) and dihydroartemisinin-piperaquine (DP) have been widely shown to be highly efficacious and well tolerated while maintaining a reduced level of acquired resistance. In addition, AL and DP therapies decrease the time of gametocyte carriage to 6 and 15 days post treatment [23]. Even the decreased circulation of infectious gametocytes after ACT is a serious impediment to controlling the dissemination of malaria parasites in high transmission regions. An increased prevalence of artemisinin resistant human *Plasmodium* species could lead to a dire scenario, but would occur only if the resistant parasite population was allowed to expand through transmission [65]. Resistance containment strategies employing transmission-blocking drugs should be a high priority to preserve the gains made with ACTs. AL and DP account for the majority of ACTs used in malaria endemic regions [1] and would be ideal to establish the proof-of-concept that BKI-ACT co-therapy can stop transmission. This approach is conceptually innovative in pairing ACT, which treats the initial malaria infection and symptoms, with a BKI as a partner drug to reduce infection of the arthropod vector, and thereby provide a community-wide benefit. To the best of our knowledge, none of the currently approved human antiparasitic drugs are administered to a patient in order to control parasites in a subsequent host, rather than strictly benefitting the individual taking the drug. Combining BKIs with ACT could help fill a current gap in malaria therapy in the form of stopping malaria transmission while alleviating the illness.

### 6.3. Slow Release Formulation of BKIs to Achieve Steady State Therapeutic Concentrations

When agents that attack molecular targets expressed in the arthropod host are administered to mammals, their transmission-blocking efficiency requires the drug’s blood concentration to be at or above a therapeutic level when a mosquito bite takes place [54]. Due to the extended presence of viable infectious gametocytes in the human host, the inhibitor is required to be non-toxic, long lasting, and effective at therapeutic doses to permit ≥28 days exposure in humans. Hence, development of a drug delivery platform that can provide sustained release of BKI into the bloodstream and maintain effective blood levels for ≥28 days after treatment would be an essential public health tool for malaria transmission control. Earlier studies have demonstrated that, of several delivery vehicles available, partition-optimized, single emulsion particles are an ideal system for incorporation and sustained release of amphiphilic BKIs [66]. Other administration formulations can be explored, including a single dose, long-acting injectable, a trans-mucosal gel that children can put under their tongue, or oral nano-suspensions that would be taken up by mucosal cells for slow release. Gummies with delayed release formulations or transdermal patches are also possible delivery systems [67,68,69,70,71,72]. Although not established, we speculate that the ideal dosing target would be once a month, with a maximum frequency of once in every one to two weeks [15]. An integration of BKIs’ pharmacokinetic properties and tissue localization profiles with that of drug formulation particles (nanocrystal or nanoparticles) or other pharmaceutically acceptable excipients could be used to develop a platform for long-acting drug release at safe and effective concentrations. Drug dose combination data can be matched with preclinical ADME and toxicology data, using pharmacokinetic modeling tools, to determine the ideal human dosage. Feasibility studies are also needed to understand the ideal parameters for acceptance and establishment of the proper target product profile.

BKIs can also be co-formulated with a blood stage antimalarial drug to develop a system to slowly release both the transmission-blocking BKI and the antimalarial to maintain effective blood levels for 1 to 3 months. We suggest formulating this combination drug as a monthly or bi-monthly oral gel using an extended-release, drug-combination nanoparticle (DcNP) formulation for prophylactic administration in East and sub-Saharan Africa. A fixed-dosage drug combination administered once a month would improve patient adherence and provide another basis for malaria control and eradication. The blood stage antimalarial drug component of the proposed combination therapy would be useful in reducing the number of clinical malaria cases over time. Since the proposed drug combination would be formulated for prophylactic use only, this would minimize the chance for acquired resistance to the blood stage antimalarial because the drugs would attain concentrations effective for combating the small number of parasites that are often present in asymptomatic individuals. Intervention strategies focused on depleting the malaria parasite reservoir and preventing continuous transmission of infectious parasites to mosquitoes would be essential for effective control and subsequent eradication.

### 6.4. Human Administration of BKIs for Transmission Blocking: Pros and Cons

Most current antimalarial drugs were developed with the single goal of rapidly and reliably alleviating malaria blood stage symptoms [73], with limited efforts to target the sexual stages of the *Plasmodium* life cycle that mediate transmission. These stages develop late in the human disease process, with the appearance of gametocytes about 4 to 15 days after the onset of symptoms, depending on the *Plasmodium* species [74]. While BKIs are not candidates for alleviating disease symptoms, they address the important adjunct challenge of reducing malaria risk in communities by reducing transmission from infected people.

Moreover, BKIs could reduce the spread of resistant mutants when combined with an existing antimalarial treatment regimen. Ten to 100 gametocytes are needed to establish a mosquito infection, even for the most effective malaria vector, *Anopheles gambiae* [75]. By inhibiting essential, early steps in the sexual stages of parasite development, the BKIs would affect the low number of gametocytes with high efficiency to prevent infection of mosquitoes with either wild-type or drug resistant malaria parasites [54]. Since exflagellation occurs in the mosquito gut within 10 to 30 min after a blood meal, the pace is likely too rapid for metabolism of a BKI to occur [54]. Furthermore, the probability of resistance emergence would be relatively low in this case, as opposed to the possibility for emergence if the asexual stage was targeted, since the number of parasites the BKI is acting on is much lower than the up to 10^9^ or 10^13^ asexual parasites per infected individual before drug treatment.

One downside of BKIs is that they do not directly benefit the humans to whom they are being administered. The physiological events that BKIs block occur within the mosquito [54], therefore, the sole purpose of administering them to humans would be to transfer the drug to mosquitoes during blood meals. Successful experiments in mice using BKI-1 and BKI-1294 (Figure 2) have demonstrated that this approach has potential promise [54,55]. However, malaria patients taking BKIs would experience no direct benefit, while being exposed to potential risks. One could counter this argument by saying that the benefit to the community would also benefit the individual and reduce the chance of the individual being re-infected with malaria. Considering the compound has to be taken as multiple doses or delivered as a long-acting depot to be present whenever gametocytes are ingested by mosquitoes, it seems unlikely that a person would take repeated doses or consent to a depot administration that benefits the community rather than the person themself. Though the risk of adverse events is anticipated to be extremely low, ethical concerns similar to those for a transmission-reducing malaria vaccine should be considered [76]. Finally, treating patients with BKIs to reduce malaria transmission would require expensive and time-consuming clinical trials before a drug could be brought to market. Taken together, these issues make the development of BKIs for clinical use a challenging proposition.

### 6.5. BKI Delivery to Mosquitoes

The above issues could be circumvented by devising a way to deliver BKIs directly to mosquitoes. Chemical agents (compounds, drugs, insecticides, etc.) can be administered to mosquitoes via several potential routes. Mosquito eggs, larvae, and pupae develop in fresh water, and can be treated during this water exposure [77]. Disadvantages of water treatment include concerns that large quantities of the chemical agent could be required and treating mosquito larvae would harm or contaminate other organisms that feed on them. In the case of adult mosquitoes, compounds can be administered via contact with a treated substrate such as an LLIN, assuming the drug is able to cross the insect cuticle. Finally, adult mosquitoes can ingest chemical agents during feeding. Only female *Anopheles* mosquitoes take blood meals, but both females and males feed on nectar. This behavior has been exploited to develop “attractive toxic sugar baits” (ATSBs), which are sugar water feeding stations laced with an insecticidal chemical. For example, adding permethrin to sugar water feeders effectively kills adult female *A. gambiae* [78].

**Figure 4:**
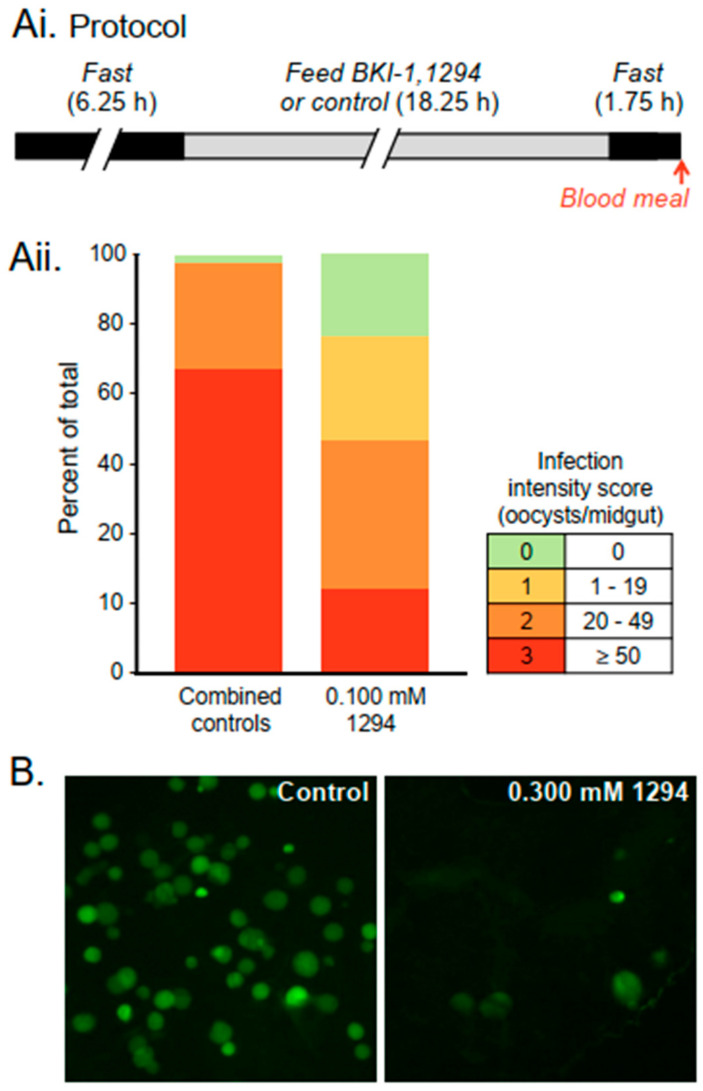
Pre-feeding mosquitoes with BKI-1294 reduced *Plasmodium* infection following an infected blood meal. (**Ai**) Experimental protocol for *P. berghei* experiment. Mosquitoes were fasted, given ad lib access to feeders containing 10% dextrose (control), or 10% dextrose with solvent (control), BKI-1 or BKI-1294, fasted again, and then provided an infected blood meal; (**Aii**) Infection intensity is shown 11 days after the infected blood meal in controls and mosquitoes pre-fed with 0.100 mM BKI-1294. Oocysts per midgut were scored using the semi-quantitative scheme shown and color-coded to highlight differences between groups. Infection intensity in mosquitoes pre-fed with 0.075 or 0.100 mM BKI-1 or 0.075 mM BKI-1294 (*n* = 20/group, data not shown), did not differ from controls (*n* = 40). In contrast, pre-feeding with 0.100 mM BKI-1294 (*n* = 20) significantly reduced infection intensity versus controls (*p* < 0.0001, Fisher’s exact test, Mann–Whitney test, 1-tailed). (**B**) Images of GFP-labeled oocysts on mosquito midguts in a similar experiment using transgenic *P. falciparum*. Mosquitoes were pre-fed with 0.300 mM BKI-1294, with oocysts counted 9–10 days after the infected blood meal. Pre-feeding with 0.100 or 0.300 mM BKI-1294 (*n* = 65–71/group) significantly reduced the number of oocysts/midgut as compared with controls (*n* = 122) (*p* = 0.0002, Fisher’s exact test, Mann–Whitney test, 1-tailed, data not shown).

On the basis of this previous work, we tested whether *Anopheles* mosquitoes would feed on sugar water laced with BKIs and, if so, whether BKI ingestion reduced infection when the mosquitoes subsequently ingested blood containing infective gametocytes. Previous studies have shown that ingestion of BKIs (via a blood meal) had no deleterious effects on mosquitoes [54,55], unlike ivermectin [15]. This is an important advantage of BKIs over ivermectin. Preliminary results indicated that pre-feeding mosquitoes with BKI-1294 in sugar water inhibited the infection of *A. stephensi* by *P. falciparum* or *P. berghei*, as quantified by the number of oocysts that developed on the midgut. Concentrations of 0.100 or 0.300 mM BKI-1294 in sugar water significantly reduced infection levels in mosquitoes as compared with the controls, whereas lower concentrations of BKI-1294 and all tested concentrations of BKI-1 provided no protection (Figure 4). BKI-1294 is modified to reduce metabolic breakdown, which could underlie its effectiveness [55]. BKI-1294 caused both an increase in the percentage of uninfected mosquitoes and a decrease in the mean number of oocysts per midgut in the mosquitoes that did become infected. In this study, BKI pre-feeding never reduced infection to zero in populations of mosquitoes taking infected blood meals. More experiments are needed, but the results provide a proof-of-principle for delivering BKIs directly to mosquitoes, rather than via mammals, to reduce malaria transmission (Figure 4).

Several factors could influence the extent to which an ingested BKI is effective in mosquitoes. First, the compound could be absent or at inadequate levels at the location and time of the *Plasmodium* events that require CDPK4, CDPK1, and PKG activity. This could result from an inadequate concentration of the compound in the sugar water or inadequate ingestion by the mosquitoes, perhaps due to a deterrent effect on feeding [79]. We observed reduced feeding when Dimethyl Sulfoxide (DMSO) was used as the solvent but did not see this effect with Tween80 and ethanol at the concentrations tested. Similarly, we observed previously, in an oral dosing study, that BKI-1294 can deter ingestion by mice even when mixed with honey [80]. It would be informative to determine the upper limit of palatability of BKI-1294 and its associated solvent. If higher concentrations of BKI-1294 deter sugar water intake by mosquitoes, potential remedies include chemical modification of the compound to enhance palatability, or addition of fruit or flower scents to the solution, as has been done in ATSB studies [81].

A second factor that could influence the effectiveness of ingested BKIs is metabolic breakdown. We showed previously that, in the mammalian liver, BKI-1 appeared to be metabolized predominantly by hydroxylation of the piperidine group [55]. Iterative modification of BKI-1 and optimization by methylation of the piperidine ring to produce compound BKI-1294 (Figure 2) resulted in over three-fold increases in the blood level and area under the curve, and a longer clearance time in mice [55]. This could be due to better protection of the site of metabolism and, likewise, could have made BKI-1294 more stable and effective than BKI-1 in these experiments. Future studies could examine the metabolism specifically in mosquitoes, to suggest modifications to optimize exposure of BKIs for sugar feeding applications.

Finally, aspects of the sugar feeding protocol, such as the duration of time mosquitoes are provided access to the feeders and the time between BKI ingestion and a blood meal, could affect a compound’s ability to reduce infection. These factors could be investigated in the future, and other compounds in the BKI series [55] could be found to be more effective than BKI-1294 in reducing infection when ingested.

One overarching challenge to the BKI feeding approach is the feasibility of delivering BKIs to mosquito populations at coverage levels sufficient to reduce malaria transmission in real world communities. As mentioned above, other potential modes of delivery include treating water sources or surfaces such as LLINs. Regardless, among TRIs being considered as part of the overall malaria control arsenal, treating mosquitoes to interrupt the *Plasmodium* life cycle deserves further consideration.

## 7. Conclusions

Successful development and clinical use of malaria transmission blocking drugs must overcome several challenges, including regulatory and safety issues, engagement of populations at risk, education and motivation for use, and epidemiologic and economic factors. Direct engagement with people in malaria-endemic regions based on durable alliances with stakeholders and community leaders is essential to implement widespread acceptance. Similarly, improving community education on the benefit of a malaria transmission blocking medication would be necessary for reshaping perceptions, sociocultural beliefs, and understanding to overcome conspiratorial deterrence. Whatever the challenges, the potential epidemiological impact of deployment of a transmission-blocking drug in malaria-endemic areas should include a considerable decrease in clinical malaria cases, direct economic benefits, and eventual eradication. Increasing interest in insecticides that address mosquito resistance and the identification of new molecules as potential transmission-blocking therapeutics emphasizes the urgency of establishing this alternative malaria control strategy [47,82,83,84]. Newly developed assay protocols are increasing the possibilities of high throughput screening of pharmaceutical libraries for potential malaria transmission-blocking leads [47,82]. These novel agents could act against a variety of potential molecular targets of transmission-blocking; especially promising are those targets previously identified by systematic functional analysis as essential regulators of malaria parasite transmission to mosquitoes [47,53]. Similarly, research studies abound on the antimalarial activities of plants against the asexual stages of *Plasmodium* [85], but few have exploited their transmission-blocking potential. Naturally derived extracts are relatively underexplored resources for novel bioactive chemical scaffolds that could accelerate the discovery of more effective antimalarial drugs capable of blocking transmission. Therefore, future ethnobotanical surveys of plants, fruits, vegetables, and fungi with reported antimalarial activities are needed to determine their transmission-blocking potential.

Few examples of drugs that act in the malaria mosquito vector exist, and therefore BKIs are unique as transmission-blocking agents, in spite of anticipated challenges for clinical development. The molecular target of BKIs, *Plasmodium* spp. CDPK4 genes, are highly conserved, as demonstrated by the inhibition by BKIs of both human and rodent malaria parasite infection of mosquitoes [54]. Therefore, if BKIs are developed, they could be useful in blocking the transmission of *Plasmodium* spp. known to cause human malaria, which would provide a significant public health benefit.
